# Complete Genome Sequence of a New Strain of *Rice yellow mottle virus* from Malawi, Characterized by a Recombinant VPg Protein

**DOI:** 10.1128/genomeA.01198-17

**Published:** 2017-11-02

**Authors:** Innocent Ndikumana, Agnès Pinel-Galzi, Denis Fargette, Eugénie Hébrard

**Affiliations:** a Research Department, Rwanda Agriculture Board, Kigali, Rwanda; b Interactions Plantes Microorganismes Environnement, Institut de Recherche Pour le Développement, Cirad, Université Montpellier, Montpellier, France

## Abstract

The complete sequence of the isolate Mw10 of *Rice yellow mottle virus* was determined. Sequence comparisons revealed 8.4% to 10.7% nucleotide divergence from the published sequences, resulting in the definition of the strain S7. Importantly, a putative recombination event was identified encompassing the viral genome-linked protein involved in host adaptation.

## GENOME ANNOUNCEMENT

*Rice yellow mottle virus* (RYMV) is endemic in Africa and Madagascar. The virus was detected first in Kenya in 1966 (1) and since then in most rice-growing countries (2). The infection induces symptoms of yellowing and mottling on leaves. The infected plants show a reduction of height and fertility. RYMV causes high losses ranging from 25% to 100% depending on the virus strain, plant genotype, and growth stage of the plants (3, 4). The transmission is mediated mechanically through agricultural practices, insects, or animals (5–7). The host range of RYMV is narrow, restricted to rice and a few wild *Poaceae* (1). RYMV is a member of the genus *Sobemovirus* (8), and its genome consists of a single-stranded positive-sense RNA with five open reading frames (9). Based on phylogenetic analyses, RYMV has been classified in six major strains with a strong, spatially based diversity (10). The highest diversity is located in East Africa, the putative center of origin and diversification of RYMV (11). Here, we present the whole-genome sequence of a new strain collected in 2014 from symptomatic rice leaves in paddy fields in the Zomba District, in the south of Malawi. The infection was confirmed after serological detection by a double-antibody sandwich (DAS) enzyme-linked immunosorbent assay (ELISA) test as previously described (12). The total RNA was extracted using the RNeasy plant minikit (Qiagen). Specific retrotranscription of the viral genome was performed using the 5′ CTCCCCCACCCATCCCGAGAATT 3′ primer (11). Two overlapping fragments covering the complete RYMV genome were amplified using primers 5′ CAATTGAAGCTAGGAAAGGAG 3′ and 5′ ACTTCGCCGGTTTCGCAGAGGATT 3′ for a first fragment and primers 5′ CATGCTGGGAAAAGTGTCTG 3′ and 5′ CTCCCCCACCCATCCCGAGAATT 3′ for a second fragment (11). The sequence of the isolate Mw10 was compared to the 33 published full-length sequences using MEGA6.06 (13). The isolate Mw10 shared only 89.3% to 91.4% nucleotide identity with the other East African sequences. This isolate belongs to a new strain distinct from the lineage S4lm found in Malawi (14) and from the other strains, S4 to S6, found in neighboring Tanzania (15), and has been named strain S7. The sequence of the isolate Mw10 was analyzed using the Recombination Detection Program version 4.0 (RDP4) (16). The strain S7 is mainly related to the strain S6, and shared its characteristic insertion-deletion polymorphisms in the coat protein gene. However, a putative recombinant event was identified in the ORF2a inside the VPg domain from an unknown parent. Unexpectedly, the sequence of the central domain of the VPg protein accumulates single polymorphisms found only in resistance-breaking genotypes of RYMV (17, 18). Also surprising, the amino acid at position 49 involved in host adaptation is neither a glutamic acid nor a threonine, but a glutamine. This unique feature is under investigation through experimental and phylogeographic studies, and the recombination breakpoints will be assessed from a larger corpus of full sequences.

### Accession number(s).

This genomic sequence has been deposited in GenBank under the accession no. MF989228.
